# Reduced CSF Water Influx in Alzheimer’s Disease Supporting the β-Amyloid Clearance Hypothesis

**DOI:** 10.1371/journal.pone.0123708

**Published:** 2015-05-06

**Authors:** Yuji Suzuki, Yukihiro Nakamura, Kenichi Yamada, Hironaka Igarashi, Kensaku Kasuga, Yuichi Yokoyama, Takeshi Ikeuchi, Masatoyo Nishizawa, Ingrid L. Kwee, Tsutomu Nakada

**Affiliations:** 1 Center for Integrated Human Brain Science, Brain Research Institute, University of Niigata, Niigata, Japan; 2 Department of Molecular Genetics, Brain Research Institute, University of Niigata, Niigata, Japan; 3 Department of Psychiatry, Faculty of Medicine, University of Niigata, Niigata, Japan; 4 Department of Neurology, Brain Research Institute, University of Niigata, Niigata, Japan; 5 Department of Neurology, University of California Davis, Davis, California, United States of America; Nathan Kline Institute and New York University School of Medicine, UNITED STATES

## Abstract

**Objective:**

To investigate whether water influx into cerebrospinal fluid (CSF) space is reduced in Alzheimer’s patients as previously shown in the transgenic mouse model for Alzheimer’s disease.

**Methods:**

Ten normal young volunteers (young control, 21-30 years old), ten normal senior volunteers (senior control, 60-78 years old, MMSE ≥ 29), and ten Alzheimer’s disease (AD) patients (study group, 59-84 years old, MMSE: 13-19) participated in this study. All AD patients were diagnosed by neurologists specializing in dementia based on DSM-IV criteria. CSF dynamics were analyzed using positron emission tomography (PET) following an intravenous injection of 1,000 MBq [^15^O]H_2_O synthesized on-line.

**Results:**

Water influx into CSF space in AD patients, expressed as influx ratio, (0.755 ± 0.089) was significantly reduced compared to young controls (1.357 ± 0.185; p < 0.001) and also compared to normal senior controls (0.981 ± 0.253, p < 0.05). Influx ratio in normal senior controls was significantly reduced compared to young controls (p < 0.01).

**Conclusion:**

Water influx into the CSF is significantly reduced in AD patients. β-amyloid clearance has been shown to be dependent on interstitial flow and CSF production. The current study indicates that reduction in water influx into the CSF may disturb the clearance rate of β-amyloid, and therefore be linked to the pathogenesis of AD.

**Trial Registration:**

UMIN Clinical Trials Registry UMIN000011939

## Introduction

The brain lacks conventional lymphatics for clearing interstitial fluid of solutes not absorbed across capillaries. The cerebrospinal flow (CSF) system has long been suggested to play a role equivalent to the systemic lymphatic system. Nevertheless, the role of CSF as the brain’s lymphatic system has not received much attention. Recently, however, the classic CSF circulation theory where CSF is almost exclusively produced by choroid plexus is found to be incomplete. It is now understood that influx from the peri-capillary (Virchow-Robin) space into the CSF system, classically referred to as interstitial flow, plays a major role in CSF production [1–3]. Furthermore, a number of studies have now shown that this interstitial flow plays a critical role in the clearance of β-amyloid [4–8]. Prealbumin (transthyretin) in the CSF has been identified to play the role of chaperon to β-amyloid, and prevents β-amyloid's natural tendency to aggregate and form plaques [9]. It is highly conceivable, therefore, that disturbance of interstitial flow may play a significant role in the pathogenesis of senile plaque (SP) formation.

Water homeostasis of the Virchow-Robin space and, therefore, interstitial flow is regulated by aquaporin-4 (AQP-4) [2,3,10], an isoform of water channels abundant in the brain. Using JJ vicinal coupling proton exchange (JJVCPE) imaging [11,12], a novel non-invasive magnetic resonance imaging (MRI) method capable of tracing exogenously applied substrates in a manner similar to positron emission tomography (PET), we had previously demonstrated that water influx into the CSF system is significantly reduced in senile plaque bearing transgenic AD model mice to the extent similar to AQP-4 knockout mice [12,13]. Using PET, we confirmed in this study that water influx into CSF is also reduced in AD patients.

## Materials and Methods

### Subjects

**Fig 1:**
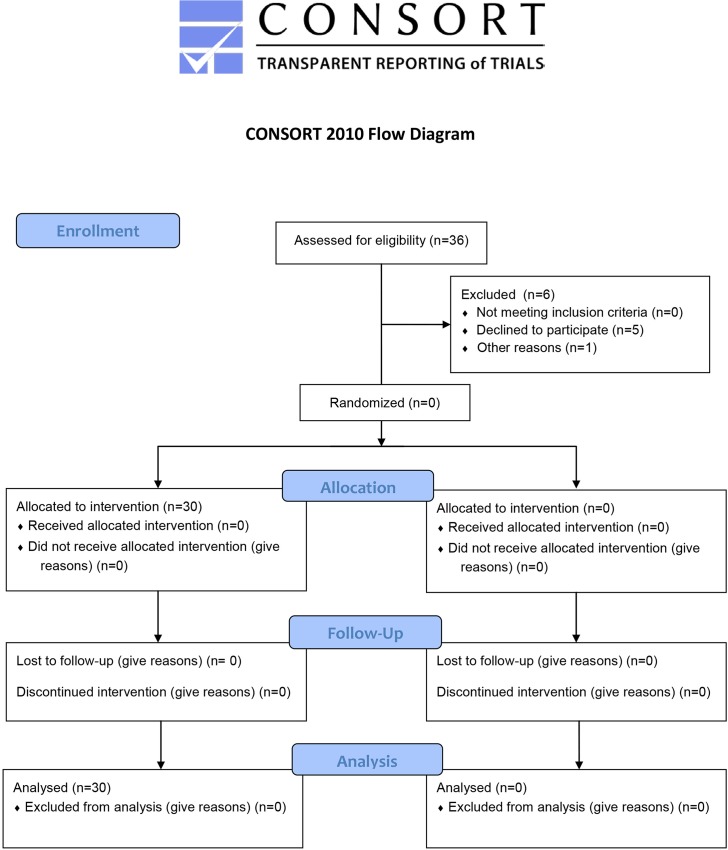
Consort Flow Diagram providing details of participant enrolment.

Ten normal young volunteers (10 males, ages 21–30 years), ten normal senior volunteers (7 males and 3 females, ages 60–78 years), and ten Alzheimer’s disease (AD) patients (6 males and 4 females, ages 59–84 years) participated in this study. Participant recruitment was completed between October 1, 2013, and April 4, 2014 (Fig 1). All volunteers were free of any significant medical conditions such as hypertension, diabetes, chronic pulmonary diseases, and were not taking any prescribed, over the counter, or herbal medications. AD patients were recruited from the Memory Clinic, Niigata University Hospital. The diagnosis of AD was determined based on DSM-IV criteria by neurologists specializing in dementia. The neurologists further determined whether the potential subject had decision making capacity to consent for the study. Age-matched senior volunteers were assessed to have no functional and no cognitive impairment (Mini-Mental State Examination (MMSE) score ≥29), and had no neurological disease. In compliance with the Institutional Review Board of University of Niigata, the study protocol was explained in detail to all potential subjects, or their proxy where appropriate. Written informed consent was obtained from all subjects or, where the Alzheimer’s subject was deemed to have no decision making capacity to consent for the study, by his/her proxy. All imaging studies were performed between October 2013 and April 2014. The study was approved by the Ethics Review Board of the Internal Review Board of University of Niigata. The project was registered at the UMIN Clinical Trials Registry as UMIN000011939 (http://www.umin.ac.jp/ctr/index.htm). Participants were provided with contact names and telephone numbers in the event of any adverse event related to the study. All participants were followed up within one month of the study to further confirm the absence or occurrence of adverse events related to the study, not otherwise self-reported by the study participant or proxy to the study coordinator. Alzheimer’s patients are additionally followed up annually at the Memory Clinic.

### PET imaging

PET imaging was performed using a combined PET/CT scanner (Discovery ST Elite, GE Healthcare, Schenectady NY, USA) with a 15 cm field of view (FOV) positioned in the region of the cerebrum. To correct for photon attenuation, low-dose CT imaging was acquired in helical mode. The subject’s head rested on a foam cushioned headrest. A head strap was applied to minimize head movement.

1000 MBq [^15^O]H_2_O, synthesized on-line ([^15^O]CO_2_ + 4H_2_ → 2[^15^O]H_2_O + CH_4_) was injected intravenously into an antecubital vein via an automatic water injection system (AM WR01, JFE Technos, Yokohama, Japan). The system delivered a 10 ml bolus over 10 seconds at 1 ml/sec with both pre- and post- flush of an inert saline solution. Immediately after starting the administration, emission data were acquired over 20 minutes in three-dimensional list mode with a 25.6 cm axial FOV and sorted into 40 time frames (40 × 30 seconds).

All emission scans were normalized for detector inhomogeneity and corrected for random coincidences, dead time, scattered radiation, and photon attenuation. For optimization of image quality, the 40 frames of the dynamic emission scans were reconstructed using 3D-OSEM (Ordered-Subset Expectation Maximization) with 2 iterations and 28 subsets. The resultant image quality allowed manual identification of regions of interest (ROIs). For the reconstruction algorithms, the data were collected in a 128 × 128 × 47 matrix with a voxel size of 2.0 × 2.0 × 3.27mm.

### Data Analysis

The CT and PET image data were transferred to a Xeleris 1.1 workstation (GE Healthcare) for PET data analysis. Manually defined ROIs on the attenuation corrected axial images and CT images (lateral and third ventricle, cortex of occipital lobe) were used to obtain the time-activity data of the scans of each subject. The tissue activity concentration in each ROI was expressed as the standardized uptake value (SUV, g/ml), corrected for the subject’s body weight and administrated dose of radioactivity. Each tissue time activity concentration was determined by fitting the data using the following equation:

$$\text{y}\left(t\right)=y_{0}+ae^{-bt}$$

where y_0_ is the baseline SUV. Subsequently, the ratio between SUVs of ventricle and cortex was defined as the influx ratio. The numerical data were subjected to the Mann-Whitney-Wilcoxon rank sum test for group analysis using IBM SPSS version 19.5 (IBM Corporation, Armonk, NY, USA).

## Results

**Fig 2:**
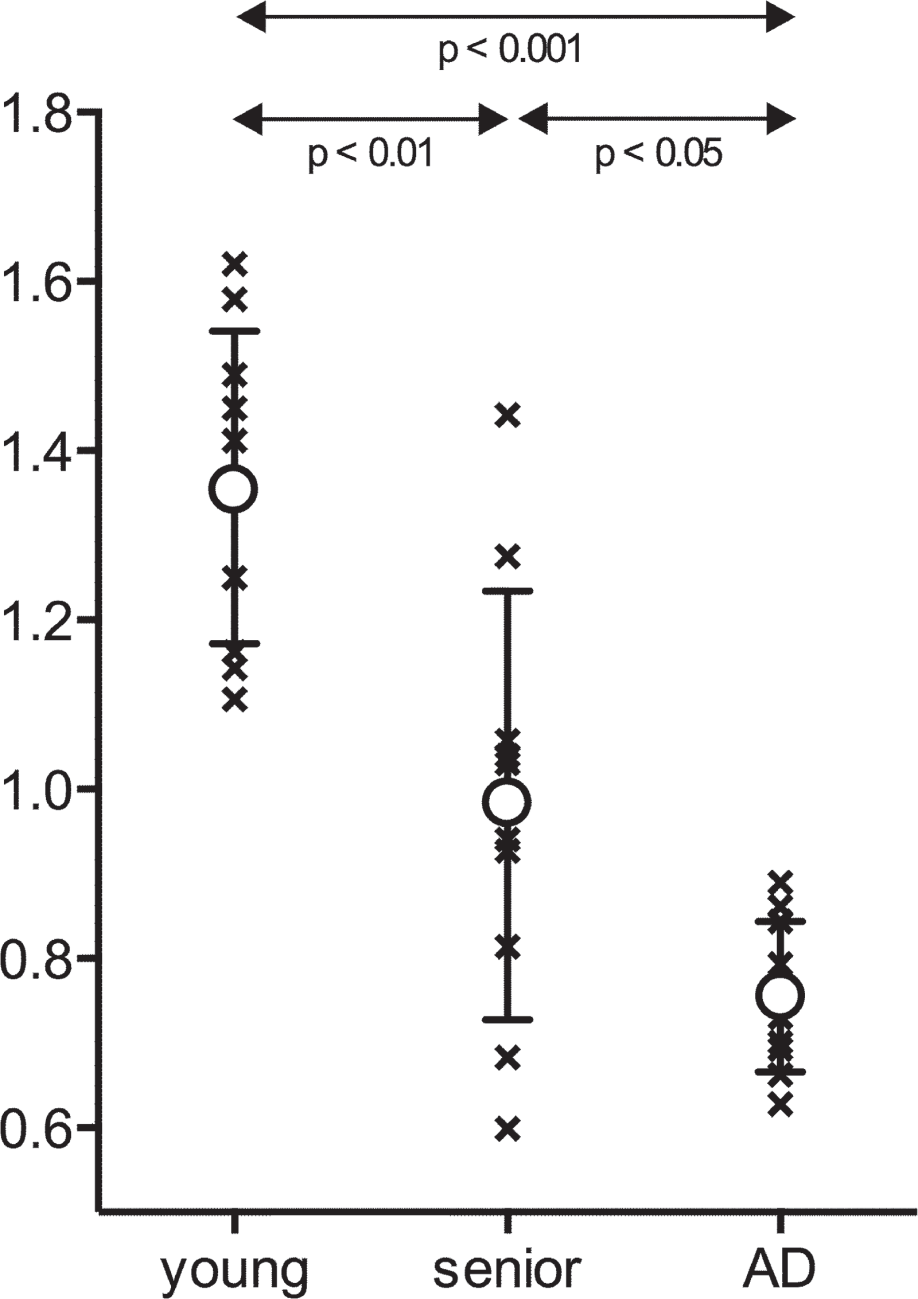
Schematic presentation of the results with mean (circle) and standard deviation (bar). Water influx into CSF space is expressed as influx ratio (IR): the ratio between the standardized uptake value (SUV, g/ml) of the ventricle to that of cortex. IR in Alzheimer’s disease patients (AD) is significantly reduced compared to both young controls (p < 0.001) and senior controls (p < 0.05), Mann-Whitney-Wilcoxon rank sum test. Note that there is no overlap in data points between AD and young controls. Reduction of influx ratio in senior controls compared to that in young control is found to be significant (p < 0.01) as well. A large range of influx ratio in senior controls suggests that the observed reduction likely represents one of the aging processes.

Results are shown in Table 1 and summarized in Fig 2. Water influx into CSF space in AD patients, expressed as influx ratio (0.755 ± 0.089), was significantly reduced compared to young controls (1.357 ± 0.185, p < 0.001) and senior controls (0.981 ± 0.253, p < 0.05). Furthermore, the influx ratio of senior controls compared to that of young controls was significantly reduced (p < 0.01) as well. The observed large range of influx ratio (0.599–1.442) in the senior controls suggested that the reduction in water influx into CSF represented an aging process.

**Table 1:** 

	Age	Influx Ratio
Young	21	1.10620
	21	1.35608
	21	1.45009
	21	1.14260
	22	1.16180
	22	1.62079
	22	1.24990
	24	1.41213
	24	1.48959
	30	1.57873
Senior	60	0.68299
	63	0.92728
	63	1.03987
	63	1.05720
	65	1.44195
	68	0.94050
	68	1.27526
	69	0.81396
	74	1.03050
	78	0.59892
AD	59	0.69261
	61	0.79321
	63	0.86105
	63	0.62796
	71	0.70088
	71	0.73065
	73	0.74510
	79	0.66232
	80	0.84389
	84	0.88935

## Discussion

Fluid-filled canals surrounding perforating arteries and veins in the brain parenchyma were recognized in the early era of modern medicine. They became known as the Virchow Robin space, so named after the first two scientists who described the structure in detail, namely, Rudolph Virchow and Charles Philippe Robin [14,15]. It was soon recognized that fluid in the Virchow Robin space may play a role similar to systemic lymphatics [4–8,16]. Studies based on modern technologies disclosed some of the physiological roles of the Virchow Robin space and interstitial fluid flow, ranging from the regulation of regional blood flow to β-amyloid clearance during sleep [17–19].

The unique anatomical features of the brain’s vascular system ensure a properly functioning blood brain barrier (BBB) and are a result of modification of the systemic circulation system. A critical property of the BBB is prevention of free water permeability across capillary walls (tight endothelium). This is in strong contrast to systemic capillaries which possess a leaky endothelium. The molecular basis of the tight endothelium is the formation of tight junctions. Various tight junction proteins such as claudin and occludin play the role of “gating control” of paracellular transport. Passive water movement across brain capillaries is strongly restricted by these tight junctions. In systemic capillaries, active water transport across endothelial cells is accomplished by the water channel aquaporin 1 (AQP-1). In brain capillaries, such water movement is totally abolished through the active suppression of AQP-1 [20]. Accordingly, water motion between capillary lumen into the peri-capillary Virchow Robin space is highly limited to restricted passive movement.

In contrast to the above situations, the peri-capillary Virchow Robin space receives constant water influx through AQP-4, another isoform of the aquaporin family, abundantly expressed in the perivascular endfeet of astrocytes [21]. Virchow Robin space water homeostasis and, hence, interstitial flow is primarily dependent on water influx through AQP-4 [2,3,10]. Accordingly, brain interstitial flow, which plays a role equivalent to the systemic lymphatic system, is now considered to be an AQP-4 dependent system [13]. The basic function of lymphatics is drainage of cellular debris that has been subjected to molecular scrutiny before being returned to venous circulation. β-amyloid has been shown to be essential for synaptic formation [22]. Disturbance in the proper clearance of β-amyloid, and negative balance between its clearance and production has been implicated to play a significant role in senile plaque formation and ultimately the pathogenesis of Alzheimer’s disease. The various components of amyloid homeostasis have become a target of therapeutic strategies [23–25]. Since under physiological conditions interstitial flow plays a critical role in the clearance of β-amyloid [4–8], disturbance in AQP-4 functionality and resultant reduction in interstitial flow could cause significant β-amyloid accumulation. Transgenic mice lacking endothelial cell expression of Agrin are shown to have reduced AQP4 while BBB remained intact. These mice have significant accumulation of β-amyloid[26].

Although it is difficult to determine whether the observed reduction in water influx into the CSF system in AD patients is the primary abnormality or merely related to β-amyloid deposits in the brain, the clear cut differences between young normal volunteers and AD patients unequivocally indicated that AD patients have significant reduction in interstitial flow. Furthermore, the theoretical possibility of contribution by choroid plexus or the leptomeningeal vasculature to the water detected in the CSF compartment cannot be totally excluded. Nevertheless, the observed large range of influx ratios in senior controls without cognitive dysfunction, strongly suggests that reduction of water influx into the CSF itself is not a sufficient factor for the pathogenesis of AD, a situation similar to senile plaque formation. There must be additional factors that eventually lead to neural death and cortical dysfunction.

Drainage of β-amyloid by interstitial flow through the Virchow Robin space into CSF is essential for maintaining proper homeostasis between β-amyloid production and clearance. The balance between β-amyloid production and its clearance appears to be vital for maintaining proper neural function. Disruption in β-amyloid homeostasis may play a critical role in the pathogenesis of senile plaque formation, and, hence, the development of Alzheimer’s disease. Assessment of the dynamic indices of production and clearance of β-amyloid in various cohort groups, including mild cognitive impairment (MCI), is warranted.

## Supporting Information

S1 TREND Checklist (PDF)
